# Inhibitors of energy metabolism interfere with antibiotic-induced death in mycobacteria

**DOI:** 10.1074/jbc.RA118.005732

**Published:** 2018-12-07

**Authors:** Bei Shi Lee, Nitin P. Kalia, Xin Er F. Jin, Erik J. Hasenoehrl, Michael Berney, Kevin Pethe

**Affiliations:** From the ‡ School of Biological Sciences, Nanyang Technological University, Singapore 637551,; the § Lee Kong Chian School of Medicine, Nanyang Technological University, Singapore 636921, and; the ¶ Department of Microbiology and Immunology, Albert Einstein College of Medicine, Bronx, New York 10461

**Keywords:** cell death, ATP, tuberculosis, antibiotics, Mycobacterium tuberculosis, antagonism, Bedaquiline, oxidative phosphorylation, Q203, Telacebec

## Abstract

Energy metabolism has recently gained interest as a target space for antibiotic drug development in mycobacteria. Of particular importance is bedaquiline (Sirturo), which kills mycobacteria by inhibiting the F_1_F_0_ ATP synthase. Other components of the electron transport chain such as the NADH dehydrogenases (NDH-2 and NdhA) and the terminal respiratory oxidase *bc*_1_:*aa*_3_ are also susceptible to chemical inhibition. Because antituberculosis drugs are prescribed as part of combination therapies, the interaction between novel drugs targeting energy metabolism and classical first and second line antibiotics must be considered to maximize treatment efficiency. Here, we show that subinhibitory concentration of drugs targeting the F_1_F_0_ ATP synthase and the cytochrome *bc*_1_:*aa*_3_, as well as energy uncouplers, interfere with the bactericidal potency of isoniazid and moxifloxacin. Isoniazid- and moxifloxacin-induced mycobacterial death correlated with a transient increase in intracellular ATP that was dissipated by co-incubation with energy metabolism inhibitors. Although oxidative phosphorylation is a promising target space for drug development, a better understanding of the link between energy metabolism and antibiotic-induced mycobacterial death is essential to develop potent drug combinations for the treatment of tuberculosis.

## Introduction

Simultaneous administration of several drugs is the cornerstone of tuberculosis (TB)^2^ treatment. This multipronged strategy constrains the selection of antibiotic resistance and shortens treatment time. The current first-line regimen consists of the administration of both rifampicin and isoniazid for 6 months, with the addition of ethambutol and pyrazinamide for the first 2 months (1). Despite being selected from a limited pool of antituberculosis drugs more than 50 years ago, this combination remains very effective with a relapse rate of less than 2% at 2 years after treatment (2). The implementation of fixed-dose therapy and directly observed therapy programs have also contributed significantly to improve the TB cure rate. Nevertheless, the emergence of drug-resistant strains is becoming increasingly common worldwide (3–5). Multidrug resistant (MDR) and extensively drug-resistant (XDR) tuberculosis accounted for more than 5% of all global cases of tuberculosis in 2016 (4). Clinical management of M/XDR tuberculosis is further complicated by the absence of a rational drug combination (6).

The recent approval of bedaquiline and delamanid brought new solutions to curb the epidemic of drug-resistant tuberculosis. However, in the absence of potent companion drugs, the emergence of resistance to bedaquiline and delamanid has been observed less than 3 years after market approval (7, 8). These two drugs are particularly interesting because they represent novel classes of antibiotics that inhibit energy metabolism in mycobacteria. Bedaquiline inhibits oxidative phosphorylation by targeting the F_1_F_0_ ATP synthase (9), whereas delamanid and related nitroimidazoles inhibit respiratory cytochromes by intracellular release of nitric oxide (10).

Energy metabolism has gained a lot of interest as a target space for drug development against mycobacteria. Several small molecules targeting various components of the pathway have been recently identified, revealing the sensitivity of this pathway to chemical inhibition. For instance, Q203 is a clinical-stage drug candidate that targets the primary terminal oxidase: cytochrome *bc*_1_:*aa*_3_ in mycobacteria (11). In addition, numerous other preclinical-stage drugs targeting the cytochrome *bc*_1_:*aa*_3_ (12–14), the type II NADH dehydrogenases (15–17), menaquinone synthesis (18, 19), and the F_1_F_0_ ATP synthase (20) were also identified.

Given the rate at which drugs targeting energy metabolism are discovered, the investigation of their interaction with classical first- and second-line anti-TB drugs becomes essential. This is particularly important in light of an emerging model that links antibiotic-induced death in bacteria to a pathway involving dysregulation of central metabolism and energetic pathways (21, 22). Mounting evidence supports the involvement of the oxidative phosphorylation (OxPhos) pathway in inducing bacterial death (21, 22); however, it is not clear whether the findings made in rapidly growing bacteria are translatable to mycobacteria.

In this study, we demonstrated that subinhibitory doses of drugs targeting energy metabolism inhibit the bactericidal potency of isoniazid and moxifloxacin. Protection against isoniazid- and moxifloxacin-induced mycobacterial death was achieved with drugs targeting the F_1_F_0_ ATP synthase, the terminal oxidases, or the maintenance of the transmembrane electrochemical gradient. The bactericidal potency of isoniazid and moxifloxacin correlated with a transient increase in intracellular ATP that was abrogated by drugs targeting energy metabolism. This study highlights the complexity of antibiotic-induced death in mycobacteria and advocates for a better understanding of this fundamental aspect to aid the development of rational drug combination for the treatment of tuberculosis.

## Results

### Bedaquiline inhibits the bactericidal potency of classical antibiotics at subinhibitory concentrations

**Figure 1.**
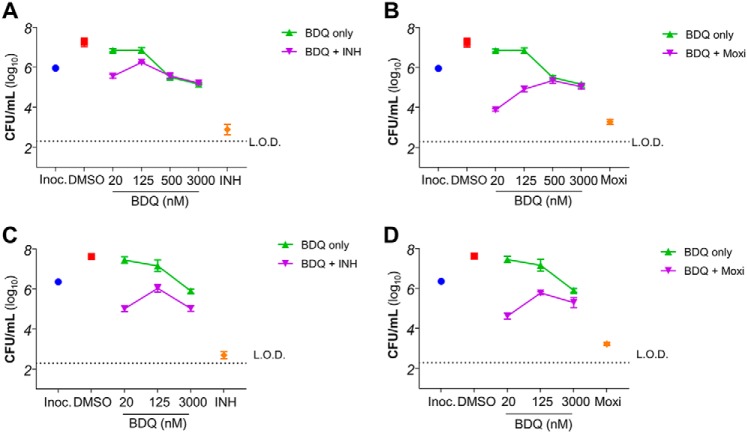
**Bedaquiline disrupts the killing efficacy of isoniazid and moxifloxacin in mycobacteria.** *A*, effect of bedaquiline (BDQ) on INH-induced killing after 3 days of incubation in *M. bovis* BCG. INH was used at 625 nm, whereas BDQ was used at 20, 125, 250, and 3000 nm. The *blue circle* represents the starting inoculum (*Inoc.*) used in the assay. The *red square* represents the effect of DMSO vehicle on bacteria viability. *B*, effect of BDQ on moxifloxacin (Moxi)-induced killing after 3 days of incubation in *M. bovis* BCG. Moxi was used at 250 nm, whereas BDQ was used at 20, 125, 250, and 3000 nm. *C*, effect of BDQ on INH-induced killing after 3 days of incubation in *M. tuberculosis* H37Rv. INH was used at 750 nm, whereas BDQ was used at 20, 125, and 3000 nm. *D*, effect of BDQ on Moxi-induced killing after 3 days of incubation in *M. tuberculosis* H37Rv. Moxi was used at 1000 nm, whereas BDQ was used at 20, 125, and 3000 nm. *L.O.D.*, limit of detection. The experiments were performed in triplicate and repeated at least once. The data are expressed as the means ± S.D. of triplicates for each condition.

We chose to study the interaction between inhibitors of energy metabolism with isoniazid and moxifloxacin, which are key drugs for the management of pan-susceptible and MDR tuberculosis, respectively. The bactericidal activity of both isoniazid and moxifloxacin is well-described. Here, we confirmed that isoniazid and moxifloxacin induce a time- and concentration-dependent killing in *Mycobacterium bovis* BCG, a surrogate nonpathogenic mycobacterium classically used for anti-TB drug testing or drug screening (23). Isoniazid and moxifloxacin reduced bacterial number by several orders of magnitude in 3 days (Fig. 1). To test the role inhibitors of energy metabolism play in the cell death pathway triggered by the administration of these classical antibiotics, co-treatment experiments were performed. Bedaquiline was supplemented into cultures treated with isoniazid or moxifloxacin, and treatment efficacy was compared. We observed that the killing efficacy of isoniazid or moxifloxacin was affected by the presence of bedaquiline. When bedaquiline was used at approximately a concentration giving half-maximal inhibition (MIC_50_; 125 nm), a concentration at which the drug alone had limited effect on mycobacterial growth (Fig. 1, *A* and *B*), it drastically inhibited the bactericidal potency of isoniazid and moxifloxacin by several orders of magnitude (Fig. 1, *A* and *B*). Even the presence of a low concentration of bedaquiline (20 nm; ∼1/5 its MIC_50_) lowered the killing efficacy of isoniazid and moxifloxacin (Fig. 1, *A* and *B*).

This rescue effect by bedaquiline was unexpected because the mechanism of action of isoniazid (drug targeting mycolic acid synthesis) and moxifloxacin (drug targeting DNA gyrase) are unrelated. Their only commonality, their bactericidal effect in mycobacteria, is thus brought into the limelight, suggesting that the OxPhos pathway may play a role in antibiotic-induced cell death. Similar results were obtained in *Mycobacterium tuberculosis* H37Rv (Fig. 1, *C* and *D*), showing that results obtained in *M. bovis* BCG are translatable to pathogenic *M. tuberculosis*.

### Other inhibitors of energy metabolism protect mycobacteria from antibiotic-induced death

**Figure 2.**
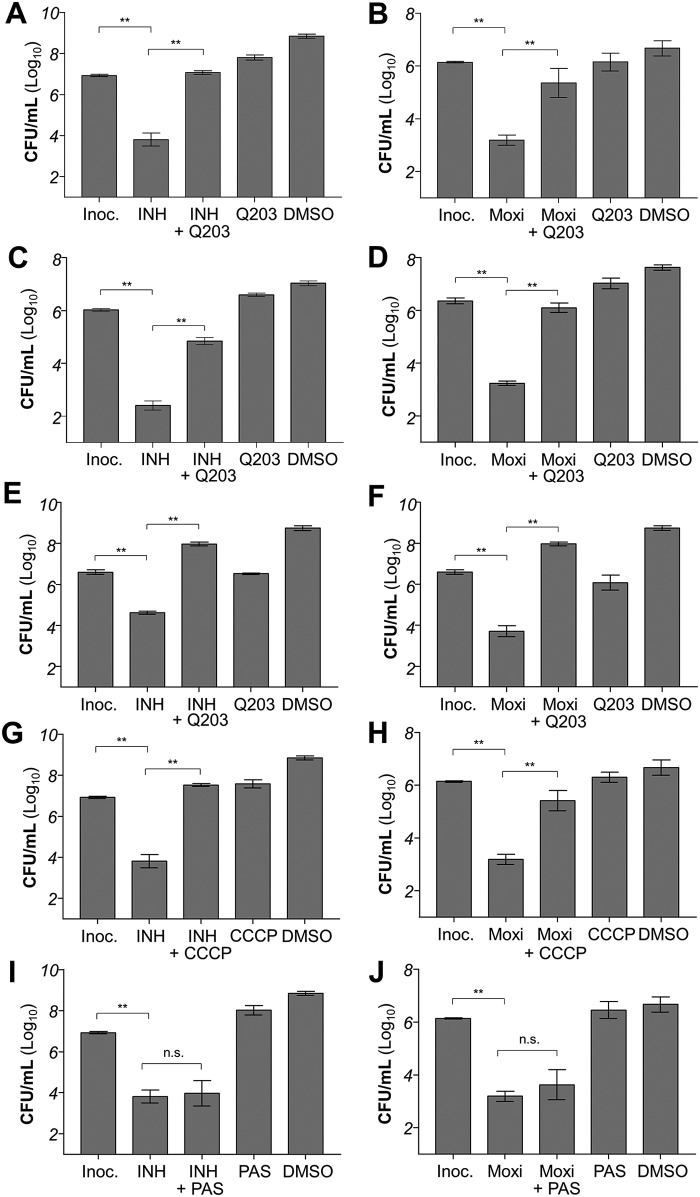
**Inhibitors of energy metabolism interfere with the mycobactericidal potency of isoniazid and moxifloxacin.** *A*, effect of Q203 on INH-induced killing after 3 days of incubation in *M. bovis* BCG. INH was used at 625 nm, whereas Q203 was used at 6.25 nm. *Inoc.*, starting inoculum size. *DMSO*, effect of the DMSO solvent control on bacterial viability. *B*, effect of Q203 on Moxi-induced killing after 3 days of incubation in *M. bovis* BCG. Moxi was used at 250 nm, whereas Q203 was used at 6.25 nm. *C*, effect of Q203 on INH-induced killing after 3 days of incubation in *M. tuberculosis* H37Rv. INH was used at 700 nm, whereas Q203 was used at 5 nm. *D*, effect of Q203 on Moxi-induced killing after 3 days of incubation in *M. tuberculosis* H37Rv. Moxi was used at 1 μm, whereas Q203 was used at 5 nm. *E*, effect of Q203 on INH-induced killing after 3 days of incubation in *M. bovis* BCG Δ*cydAB*. INH was used at 625 nm, whereas Q203 was used at 6.25 nm. *F*, effect of Q203 on Moxi-induced killing after 3 days of incubation in *M. bovis* BCG Δ*cydAB*. Moxi was used at 250 nm, whereas Q203 was used at 6.25 nm. *G*, effect of CCCP on INH-induced killing after 3 days of incubation in *M. bovis* BCG. INH was used at 625 nm, whereas CCCP was used at 12.5 μm. *H*, effect of CCCP on Moxi-induced killing after 3 days of incubation in *M. bovis* BCG. Moxi was used at 250 nm, whereas CCCP was used at 12.5 μm. *I*, effect of *para*-aminosalicylic acid (PAS) on INH-induced killing after 3 days of incubation in *M. bovis* BCG. INH was used at 625 nm, whereas PAS was used at 500 nm. There was no statistically significant difference (*p* = 0.97, Tukey's multiple comparisons test) in cfu counts between INH treatment and INH–PAS co-treatment. *J*, effect of PAS on Moxi-induced killing after 3 days of incubation in *M. bovis* BCG. Moxi was used at 250 nm, whereas CCCP was used at 500 nm. There was no statistically significant difference (*p* = 0.29, Tukey's multiple comparisons test) between Moxi treatment and Moxi–PAS co-treatment. **, *p* < 0.0001, Tukey's multiple comparisons test. The experiments were performed in triplicate and repeated at least once. The data are expressed as the means ± S.D. of triplicates for each condition.

Next, we tested whether the rescue effect caused by bedaquiline could be replicated using other drugs targeting the OxPhos pathway. We selected Q203, which is a clinical-stage drug candidate targeting the terminal respiratory oxidase cytochrome *bc*_1_:*aa*_3_ (11). Unlike bedaquiline, which is bactericidal at high dose, Q203 is bacteriostatic because of the presence of the alternate cytochrome *bd* oxidase (24). When tested at 1–6-fold its MIC_50_, Q203 protected *M. bovis* BCG (Fig. 2, *A* and *B*) and *M. tuberculosis* (Fig. 2, *C* and *D*) against the lethal action of isoniazid and moxifloxacin as well. We have previously shown that Q203 is bactericidal at low dose against mycobacteria strains deficient for the expression of the cytochrome *bd* oxidase (24). Q203 protected *M. bovis* BCG from isoniazid and moxifloxacin-induced death even in a *cydAB* knockout background (Fig. 2, *E* and *F*), suggesting that the rescue effect of Q203 is unrelated to its bacteriostatic effect. Furthermore, it was observed that even the unspecific protonophore carbonyl cyanide *m*-chlorophenyl hydrazone (CCCP) inhibits the bactericidal potency of isoniazid and moxifloxacin at subinhibitory concentrations (Fig. 2, *G* and *H*), reinforcing the notion that OxPhos inhibition and uncoupling protect mycobacteria from antibiotic-induced cell death. Conversely, a subinhibitory concentration of *para*-aminosalicylic acid, a bacteriostatic anti-TB drug targeting folate synthesis (25), does not protect mycobacteria from isoniazid- or moxifloxacin-induced death (Fig. 2, *I* and *J*). This result suggests that only inhibitors of energy metabolism have the capacity to interfere with the process of bacterial killing induced by antibiotics.

### Inhibitors of energy metabolism affects early bactericidal activity

**Figure 3.**
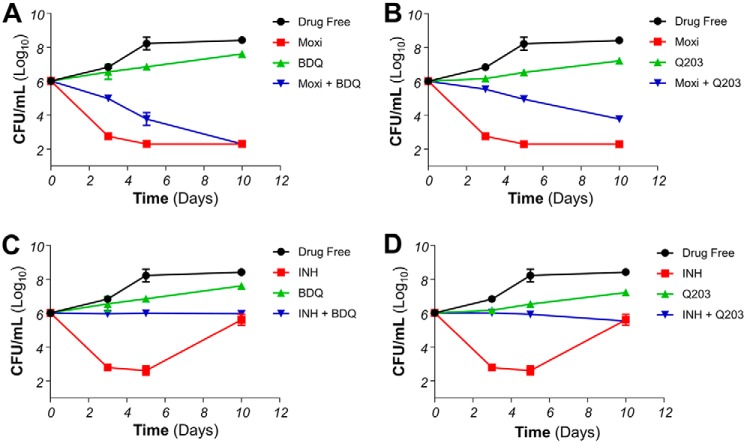
**Respiratory inhibitors affect early bactericidal activity of antibiotics in *M bovis* BCG.** *A*, effect of BDQ on moxifloxacin (Moxi)-induced killing over 10 days. Bacteria viability was determined by enumerating cfu after plating on agar plates on days 3, 5, and 10. Moxi was used at 250 nm, whereas BDQ was used at 125 nm. *B*, effect of Q203 on Moxi-induced killing over 10 days. Moxi was used at 250 nm, whereas Q203 was used at 5 nm. *C*, effect of BDQ on INH-induced killing over 10 days. INH was used at 625 nm, whereas BDQ was used at 125 nm. *D*, effect of Q203 on INH-induced killing over 10 days. INH was used at 625 nm, whereas Q203 was used at 5 nm. The experiments were performed in triplicate and repeated at least once. The data are expressed as the means ± S.D. of triplicates for each condition.

To determine whether this rescue effect persists over a length of time, kill kinetic assays were performed. Consistent with other reports, isoniazid and moxifloxacin are fast-acting bactericidal drugs, triggering more than 99.9% reduction in live bacteria count by day 5 post-antibiotic treatment (Fig. 3). The number of viable cells dropped steadily across 10 days in cultures treated with moxifloxacin (Fig. 3, *A* and *B*). Co-treatment experiments revealed that bedaquiline delayed moxifloxacin-induced death but did not prevent it. Indeed, protection was maximal after 3 days of co-incubation but diminished over time to be no longer significant after 10 days (Fig. 3*A*). Co-treatment with Q203 followed a similar trend, even though a significant degree of protection was still observed after 10 days of co-incubation (Fig. 3*B*). On the other hand, the protective effect of OxPhos inhibitors on INH-induced bacterial death persisted at least up to day 5, with no drop in cfu counts compared with day 0 observed at this time point (Fig. 3, *C* and *D*). Later time points could not be analyzed because of the rapid emergence of INH resistance as reported before (26, 27).

### ATP deregulation induced by classical antibiotics is inhibited by OxPhos inhibitors

**Figure 4.**
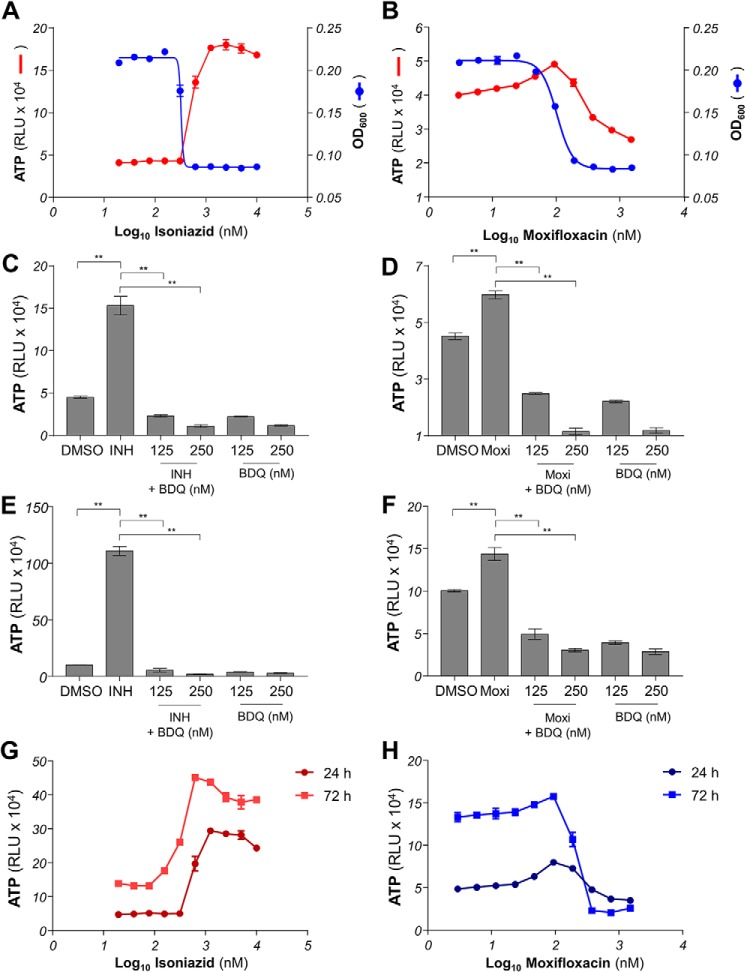
**The effect of antibiotic treatments on intracellular ATP level.** *A*, dose-response effect of INH on intracellular ATP level (*red circles*) and growth inhibition (*blue circles*) in *M. bovis* BCG. Intracellular ATP level was quantified after 24 h of incubation with respective treatments. The effect of INH on growth was determined after 5 days of incubation. *B*, dose-response effect of Moxi on intracellular ATP level (*red circles*) and growth inhibition (*blue circles*) in *M. bovis* BCG. The effect of Moxi on growth was determined after 5 days of incubation. *C*, effect of BDQ on INH-induced intracellular ATP spike in *M. bovis* BCG. INH was used at 625 nm, whereas BDQ was used at 125 and 250 nm. *DMSO*, effect of the DMSO solvent control on intracellular ATP level. *D*, effect of BDQ on Moxi-induced intracellular ATP spike in *M. bovis* BCG. Moxi was used at 100 nm, whereas BDQ was used at 125 and 250 nm. *E*, effect of BDQ on INH-induced intracellular ATP spike in *M. tuberculosis* H37Rv. INH was used at 700 nm, whereas BDQ was used at 125 and 250 nm. *F*, effect of BDQ on Moxi-induced intracellular ATP spike in *M. tuberculosis* H37Rv. Moxi was used at 200 nm, whereas BDQ was used at 125 and 250 nm. *G*, dose-response effect of isoniazid on intracellular ATP level in *M. bovis* BCG after 24 h (*dark red circles*) or 72 h (*red squares*) of incubation. *H*, dose-response effect of moxifloxacin on intracellular ATP level in *M. bovis* BCG after 24 h (*dark blue circles*) or 72 h (*blue squares*) of incubation. **, *p* < 0.0001, Tukey's multiple comparisons test. The experiments were performed in triplicate and repeated at least once. The data are expressed as the means ± S.D. of triplicates for each concentration of a representative experiment.

**Figure 5.**
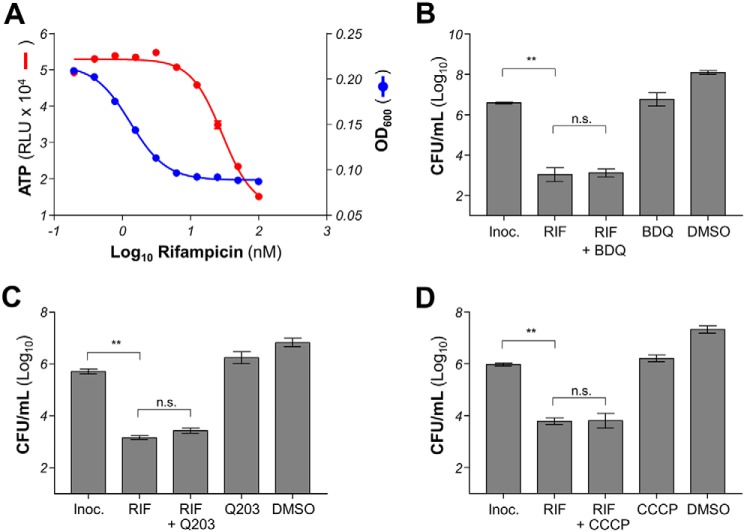
**Effect of respiratory inhibitors on rifampicin activity in *M. bovis* BCG.** *A*, dose-response effect of rifampicin (RIF) on intracellular ATP level (*red circles*) and growth inhibition (*blue circles*) in *M. bovis* BCG. Intracellular ATP level was quantified after 24 h of incubation with various concentrations of RIF. The effect of RIF on growth was determined after 5 days of incubation. *B*, effect of BDQ on RIF-induced killing after 3 days of incubation in *M. bovis* BCG. RIF was used at 50 nm, whereas BDQ was used at 125 nm. *Inoc.*, starting inoculum size. *DMSO*, effect of the DMSO solvent control on bacterial viability. There was no statistically significant difference (*p* = 0.99, Tukey's multiple comparisons test) between RIF treatment and RIF–BDQ co-treatment. *C*, effect of Q203 on RIF-induced killing after 3 days of incubation in *M. bovis* BCG. RIF was used at 50 nm, whereas Q203 was used at 6.25 nm. There was no statistically significant difference (*p* = 0.23, Tukey's multiple comparisons test) between RIF treatment and RIF–Q203 co-treatment. *D*, effect of CCCP on RIF-induced killing after 3 days of incubation in *M. bovis* BCG. RIF was used at 50 nm, whereas CCCP was used at 12.5 μm. There was no statistically significant difference (*p* > 0.99, Tukey's multiple comparisons test) between RIF treatment and RIF–Q203 co-treatment. **, *p* < 0.0001, Tukey's multiple comparisons test. The experiments were performed in triplicate and repeated at least once. The data are expressed as the means ± S.D. of triplicates for each condition.

To test whether the mycobacterial death induced by isoniazid and moxifloxacin involved a deregulation of energy metabolism that could be inhibited by bedaquiline or Q203, we analyzed the early cellular response to drug treatment by quantifying intracellular ATP levels. An increase in intracellular ATP level was observed as early as 24 h post-treatment in isoniazid- and moxifloxacin-treated mycobacteria. Isoniazid triggered an ATP spike in a pattern that was inversely proportional to its MIC curve (Fig. 4*A*), whereas the ATP response induced by moxifloxacin treatment followed a bell curve centered on the MIC_50_ of the drug (Fig. 4*B*). The increase in ATP level was higher in isoniazid-treated compared with moxifloxacin-treated mycobacteria. In both cases, the ATP burst could be dissipated by co-incubation with the F_1_F_0_ ATP synthase inhibitor bedaquiline in BCG (Fig. 4, *C* and *D*) and in *M. tuberculosis* H37Rv (Fig. 4, *E* and *F*). It was also noted that the intensity of ATP deregulation was more pronounced in *M. tuberculosis* H37Rv compared with *M. bovis* BCG. The dose-dependent intracellular ATP responses induced by isoniazid and moxifloxacin were sustained for at least 3 days (Fig. 4, *G* and *H*), suggesting that the deregulation of energy metabolism is more than a transient survival response but may also be involved in a cellular response leading to bacterial death. Interestingly, the bactericidal drug rifampicin did not trigger an ATP burst in mycobacteria (Fig. 5*A*), and inhibitors of energy metabolism did not protect against rifampicin-induced bacterial death (Fig. 5, *B–D*), showing that a putative cell-death pathway induced by mycobactericidal drugs is not universal.

## Discussion

Traditional classification of antibiotics is based on their mechanisms of action and drug–target interactions (28). However, a growing body of evidence suggests that target engagement cannot be the sole explanation of antibiotics' bactericidal action, and there is a gap in our understanding of the downstream pathways induced by bactericides that eventually lead to bacterial death (21).

Here we showed that an operative central energetics metabolism is an essential part of a pathway involved in bacterial death induced by some antibacterial drugs in mycobacteria. This is consistent with findings in *Escherichia coli*, which highlighted the role of the respiratory chain in producing reactive oxygen species in antibiotic-induced bacterial death (21). Our results showed that both isoniazid and moxifloxacin treatment induced a response resulting in a significant increase in intracellular ATP levels, an early cellular response suggesting an up-regulation of metabolic fluxes and deregulation of bioenergetics homeostasis that correlates with bacterial death. Co-incubation of such antimicrobials with energy metabolism inhibitors abolished the spike in ATP and prevented the bactericidal effect of isoniazid and moxifloxacin. Our data demonstrate that the respiratory chain, whose effect can be manipulated using respiratory inhibitors such as bedaquiline and Q203, plays a crucial role in a pathway leading to bacterial death.

It has been well-established that isoniazid and moxifloxacin are only bactericidal against actively replicating mycobacteria but are less effective against nonreplicating subpopulation (29–31). This phenomenon is in agreement with the viewpoint that the impairment in cell wall biosynthesis or DNA replication is detrimental when bacteria undergo cell division. Although one could reason that respiratory inhibitors like Q203 prevented isoniazid or moxifloxacin-induced death by inhibiting replication and mimicking the dormant state of mycobacteria, it appears that this is not the case because bedaquiline and Q203 were protective at concentrations that were insufficient to inhibit growth. Furthermore, *para*-aminosalicylic acid, a bacteriostatic anti-TB agent, does not confer said protection, thus clarifying that a bacteriostatic effect alone is not sufficient to protect mycobacteria from isoniazid- and moxifloxacin-induced death. These points combined suggest that energetics inhibitor-mediated rescue from cell death is more intricate than merely blocking bacteria replication.

Although there is strong evidence showing the key involvement of central metabolism and bioenergetics in antibiotic-induced cell death, the truth remains that our understanding of the mechanisms induced by antibiotics are largely incomplete. Our data set with rifampicin revealed the complexity of this topic, because the absence of effect of respiratory inhibitors suggests that rifampicin-induced death is independent of bioenergetics deregulation. This could be due to the presence of multiple cascades regulating stress response, and rifampicin-induced death might be regulated by another pathway which does not involve the participation of the respiratory chain. Furthermore, the deregulation of ATP homeostasis triggered by isoniazid and moxifloxacin are distinct in their profile, suggesting that their mechanisms of cell death are overlapping yet distinct.

Not only do the observations reported here contribute to the fundamental understanding of the mechanisms involved in antibiotic-induced death, it also unveils possible implications for combination therapies for tuberculosis. Although the presence of respiratory inhibitors such as bedaquiline and Q203 affect the early bactericidal activity of isoniazid and moxifloxacin *in vitro*, it is important to note that the outcome of this study does not invalidate the strategy of combining respiratory inhibitors with traditional antituberculars. First, it remains to be demonstrated that our *in vitro* observations are translatable *in vivo* and ultimately in man. As long as the concentration of the energy metabolism inhibitors are above their growth inhibitory concentrations and administered for a sufficient period of time, inhibition of antibiotic-induced bacterial death may not be seen.

Furthermore, several lines of evidence suggest that drugs targeting the oxidative phosphorylation pathway have the potential to be incorporated in a rational drug combination for the treatment of drug-resistant tuberculosis (32, 33). For instance, it has been demonstrated in a phase 2 clinical study that the addition of bedaquiline to the standard drug regimen for the treatment of pulmonary MDR-TB resulted in a faster, higher culture conversion rate, suggesting bedaquiline's treatment compatibility with drugs such as kanamycin, ofloxacin, ethionamide, pyrazinamide, and cycloserine (32). Nevertheless, significant work remains to be done to optimize dosing regimens with antibiotics having contrasting pharmacokinetics properties.

The mechanism of antibiotic-induced death is far from resolved, and the link between antibiotic action and bioenergetics remains to be deciphered at the molecular level. This presents an exciting field of research because the implication of resolving key components of a pathway triggering mycobacterial death is significant. From a practical standpoint, it gives rise to opportunities to manipulate the system and opens up a target space of great potential for drug development. By exploiting the pathogen's fundamental vulnerability, drugs targeting this pathway would promise high killing efficacy, thus improving treatment strategies against this deadly disease.

## Experimental procedures

### Strains and growth conditions

*M. tuberculosis* H37Rv, *M. bovis* BCG, and derivative strains were grown in Middlebrook 7H9 broth medium supplemented with 0.2% glycerol, 0.05% Tween 80, and 10% ADS supplement. Hygromycin B (80 μg/ml) selection was used in cultivation of *cydAB* knockout BCG strain. Prior to the start of all experiments, replicating cultures were harvested at logarithmic phase, washed to remove glycerol, and diluted to specified cell density according to specific experiments.

### Antimicrobial compounds

Q203 and bedaquiline were obtained from GVK Biosciences Private Limited. Isoniazid was obtained from Abcam. Rifampicin, moxifloxacin, CCCP, *para*-aminosalicylic acid were obtained from Sigma–Aldrich. Stock solutions of drug compounds were prepared by reconstitution in 90% DMSO. Moxifloxacin was reconstituted using 1 m NaOH in addition to 90% DMSO.

### Kill-kinetics assay

Bacteria cultures were adjusted to *A*_600_ 0.005 and dispensed into 96-well plates. Drug combinations were also aliquoted into respective wells. Total DMSO concentration in all wells were kept at 0.9%. The plates were then incubated at 37 °C for 3–10 days. Bacteria viability was determined by enumerating cfu after plating on agar plates.

### Intracellular ATP quantification

The BacTiter-Glo^TM^ microbial cell viability assay (Promega) was used for quantitation of ATP content of bacteria cultures. Bacteria cultures were adjusted to *A*_600_ 0.05 and dispensed into 96-well white plates, and drug compounds were subsequently added. Quantitation of ATP was conducted after 24 h of incubation at 37 °C.

### Growth inhibition assay

Bacteria cultures were adjusted to *A*_600_ 0.005 and dispensed into 96-well plates. Drug were aliquoted into respective wells. DMSO concentration in all wells were kept at 0.9%. The plates were then incubated at 37 °C for 5 days. The growth of culture in each well was quantified by measuring optical density at 600-nm wavelength on EPOCH 2 or Cytation 3 microplate spectrophotometers.

## Author contributions

B. S. L., N. P. K., X. E. F. J., and E. J. H. formal analysis; B. S. L. and N. P. K. validation; B. S. L., N. P. K., X. E. F. J., and E. J. H. investigation; B. S. L., M. B., and K. P. methodology; B. S. L. and K.P. writing-original draft; B. S. L., N. P. K., X. E. F. J., E. J. H., M. B., and K. P. writing-review and editing; K. P. conceptualization; K. P. supervision; K. P. funding acquisition.

## Footnotes

2 The abbreviations used are:

TB: tuberculosis
MDR: multidrug resistant
XDR: extensively drug resistant
OxPhos: oxidative phosphorylation
BCG: *Bacillus* Calmette–Guérin
CCCP: carbonyl cyanide *m*-chlorophenyl hydrazone
PAS: *para*-aminosalicylic acid
INH: isoniazid
Moxi: moxifloxacin
RIF: rifampicin
BDQ: bedaquiline.
